# Harnessing chemokines and nanocarriers to reprogram the tumor microenvironment

**DOI:** 10.1080/10717544.2026.2752676

**Published:** 2026-10-29

**Authors:** Ruixue Bai, Anya Wang, Yao Li, Yunong Li, Xueyi Song, Weimin Ni, Qian Wei, Jia Bi, Minjie Wei, Lin Zhao, Enshuo Liu, Mingyi Ju

**Affiliations:** a Department of Pharmacy, Shengjing Hospital of China Medical University, Shenyang, P.R. China; b Department of Pharmacology, School of Pharmacy, China Medical University, Shenyang, P.R. China; c Liaoning Key Laboratory of Molecular Targeted Anti-tumor Drug Development and Evaluation, China Medical University, Shenyang, P.R. China; d Department of Neurosurgery, The First Affiliated Hospital of Jinzhou Medical University, Jinzhou, P.R. China; e Endoscopy Center, Shengjing Hospital of China Medical University, Shenyang, Liaoning Province, P.R. China

**Keywords:** Nanotherapy, tumor immune microenvironment, biomimetic nanoparticles, nanoparticles, chemokine

## Abstract

Although cancer immunotherapy has significantly altered the treatment landscape for malignant tumors, the intricate and diverse tumor microenvironment (TME) still limits its effectiveness in solid tumors. The TME is a complex and heterogeneous environment, shaped predominantly by chemokines rather than being a passive consequence of various biological processes. Therefore, it is essential to comprehend and fully utilize chemokine-guided immune cell navigation in order to overcome immunological rejection and increase the effectiveness of combination cancer therapy. In this review, we begin by summarizing the developing understanding of chemokine involvement in the TME. We then look at how distinct chemokine axes direct immune cells to different tumor areas, as well as the important elements that influence these processes. In addition, we discuss recent developments in integrating chemokines or chemokine receptors with nanodelivery technologies. These systems can stabilize chemokines, prolong local retention, create sustained gradients, and use chemokine-receptor interactions for targeted delivery. Overall, this review provides an updated framework for understanding chemokine-guided tumor immune modulation, as well as insights for developing next-generation nanomedicines and chemokine-based immunotherapeutic methods.

## Graphical abstract

**Figure:**
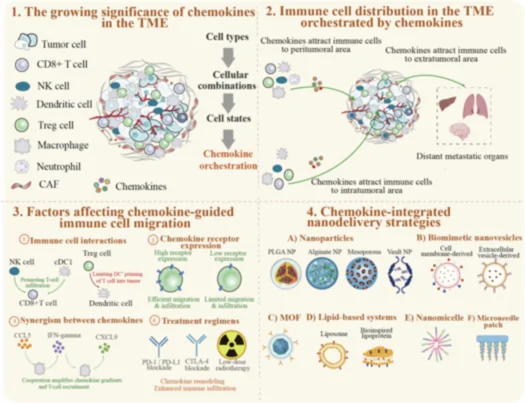

## 1. Introduction

One of the most promising and rapidly expanding areas of cancer therapy is cancer immunotherapy, which has completely changed the way tumors are treated. Cancer immunotherapy's success depends not only on whether immune effector cells are activated, but also on whether they can be recruited, kept inside, and functionally positioned in the right anatomical niches of the tumor (Galon et al. 2006; Wang et al. 2023; Chen et al. 2024). Recent research has reinterpreted chemokines as active organizers of the TME rather than merely inflammatory mediators. Chemokines are among the most basic molecular systems that control immune-cell movement and tumor location (Galli et al. 2020; Ozga et al. 2021; Märkl et al. 2022; Mempel et al. 2024). Accordingly, a comprehensive and up-to-date understanding of how chemokine networks shape the spatial distribution of immune cells – promoting their intratumoral accumulation, exclusion from tumor-cell regions, or redistribution to alternative niches – is essential for optimizing immunotherapeutic strategies.

Chemokines are being investigated more and more as potential therapeutic agents that can influence immune-cell trafficking and spatial organization to change the TME (Märkl et al. 2022). Intratumoral administration of CCL21 gene-modified dendritic cells in advanced non-small-cell lung cancer has been shown to induce tumor antigen-specific immune responses, increase CD8⁺ T-cell infiltration, and support additional combination with pembrolizumab in a phase I setting (Lee et al. 2017; Lisberg et al. 2024). However, because chemokine-mediated cell migration depends on stable, localized gradients and tissue presentation rather than just systemic availability, successful chemokine therapy necessitates more than temporary exposure (Griffith et al. 2014). Nanodelivery technologies provide a sensible answer to these restrictions by protecting labile cargos, prolonging local retention, allowing continuous release, and increasing the spatiotemporal control of immunomodulatory drugs in tumors (Martin et al. 2020; Fan et al. 2023). An implanted hydrogel reservoir containing CCL21-expressing cells enabled sustained local CCL21 secretion and enhanced the systemic antitumor efficacy of radiotherapy (Gao et al. 2024). These findings underscore the potential of prolonged intratumoral chemokine production to promote immune remodeling and improve therapeutic responses. Concurrently, local chemokine expression can create intratumoral chemokine gradients, attract CXCR3⁺ T cells, boost T-cell infiltration, and work in concert with PD-1/PD-L1 blockade, as shown by tumor-specific gene nanomedicines encoding CXCL9 or nanoparticles delivering CXCL9/10/11 plasmids (Wang et al. 2023). As a result, chemokines and nanomedicines have a mutually reinforcing effect: chemokines offer specific biological signals that attract receptor-expressing nanoparticles into tumors, while nanocarriers offer the drug delivery instruments needed to stabilize, localize, and maintain these signals. The functional complementarity between chemokines and nanomedicines suggests that their integration may unlock therapeutic benefits beyond either modality alone, warranting a systematic assessment of this emerging strategy in cancer therapy.

In this review, we first outline how the role of chemokines as key regulators of the TME has been progressively elucidated. Next, we go over how chemokines direct immune cells to specific spatial compartments, such as the extratumoral areas, invasive margin, and tumor core. The primary determinants of chemokine-mediated immune-cell dispersion are then examined. Finally, we describe new developments in the integration of nanodelivery systems with chemokines. This review attempts to provide an updated framework for comprehending how chemokine-guided spatial immune regulation can be harnessed by nanomedicine and to offer new perspectives for the development of chemokine-based therapeutic strategies and nanoformulations in cancer treatment by concentrating on studies published in the last five years.

## 2. The growing significance of chemokines in the TME

Paget proposed the ‘seed and soil’ idea in 1889, highlighting TME's role in encouraging tumor growth (Paget 1989). The term ‘tumor microenvironment’ was subsequently coined by Whiteside (Ioannides and Whiteside 1993). According to Whiteside, TME serves as a ‘fertile ground’ for malignant behaviors such as the proliferation, progression, and metastasis of tumor cells. The conventional view of cancer as a malignant cell-centric disease is rapidly being replaced by an understanding of the dependence and interaction between cancer cells and the TME. Camus and colleagues provided a comprehensive description of the immune response in primary colorectal cancers of individuals with high or low infiltrating T cell amounts (Camus et al. 2009). Their results indicate the first step towards developing the hypothesis of hot and cold tumors. Since 2015, researchers have been using the terms ‘cold tumor’ and ‘hot tumor’ to describe tumor immune status according to the degree of CD8^+^ T-cell infiltration in the TME (Sharma and Allison 2015; Haanen 2017). However, some research indicates that the number of T cells does not necessarily equate with the strength of antitumor immunity (Chen et al. 2021). The fact that most T cells that enter tumors are bystanders and cannot destroy tumor cells may be the reason for this (Simoni et al. 2018) (Figure 1).

**Figure 1.**
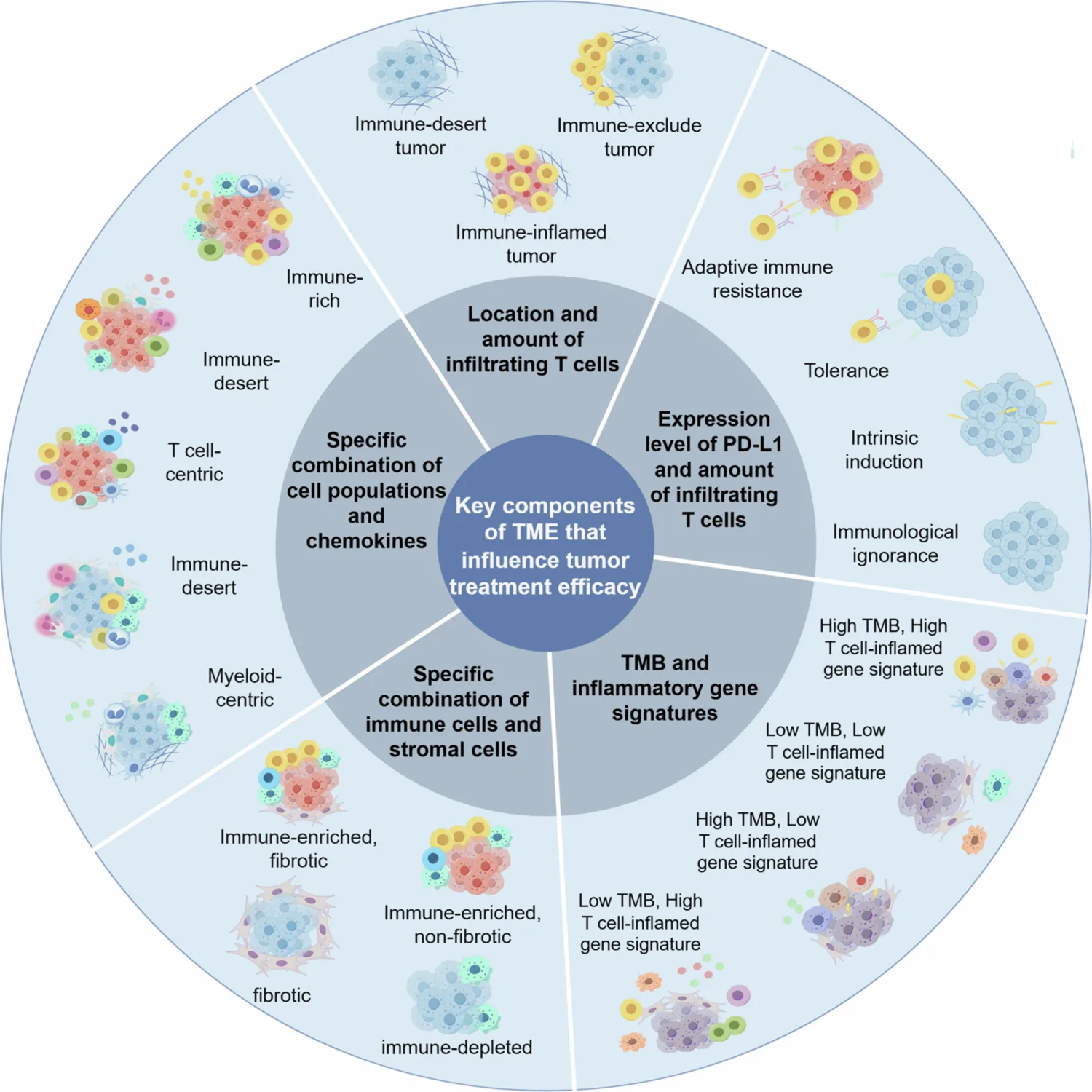
Key elements in the TME affecting cancer therapeutic efficacy. Since 2009, researchers have employed a range of experimental approaches to investigate the characteristics of TME. Over time, the influence of various components, including cell-surface receptors, monocytes, stromal cells, chemokines, and others, on the characteristics of the TME has been widely elucidated.

Based on the presence of tumor-infiltrating lymphocytes (TILs) and PD-L1 expression in melanoma patients, Michele and colleagues classified TME into four subtypes, including (I) adaptive immune resistance, (II) immunological ignorance, (III) intrinsic induction, and (IV) tolerance (Teng et al. 2015). TME subtypes defined by PD-L1 expression and immune infiltration may help predict clinical outcomes in patients with advanced non-small cell lung cancer treated with anti-PD-1/PD-L1 therapy or chemoimmunotherapy (Shirasawa et al. 2021; Sun et al. 2023). However, it has been shown that the effectiveness of PD-1/PD-L1 therapy varies significantly among individuals, with some patients showing limited or no response and others showing strong responses (Topalian et al. 2012; Le et al. 2015).

Jake O'Donnell and colleagues classified TME into four categories based on tumor mutational burden (TMB) and T-cell inflammatory gene signature through a pan-cancer genomic analysis of The Cancer Genome Atlas (O'Donnell et al. 2019). TMB is defined conceptually as the overall amount of mutations in a tumor sample (Sha et al. 2020; Jardim et al. 2021). Tumor mutations are processed into neoantigens, which are then presented on the surface of cancer cells by major histocompatibility complex proteins. T cells recognize these neoantigens and destroy the cancer cells. However, it is not always the case that a significant number of mutations will result in immunogenic neoantigens (Lauss et al. 2017), which may depend on the type of cancer (Anagnostou et al. 2022). In a retrospective cohort study, including 1678 patients, when cancer type-specific TMB was used, response rates were found to be higher in TMB-high tumors than in TMB-low tumors in virtually all cancer types. However, when a single universal TMB threshold was used, TMB-high tumors had higher response rates than TMB-low tumors in only 11 of the 16 cancer types (Valero et al. 2021). In conclusion, when using TMB as a basis for classification, the tumor type should be sufficiently taken into account. Otherwise, the usefulness of this classification is limited.

Through transcriptomic analysis of more than 10,000 cancer patients, Bagaev and associates identified 29 functional gene expression signatures (Fges) that may indicate the major functional elements of the tumor along with its stromal, immune, and other cellular populations. TME classification reveals four distinct TME subtypes conserved across 20 different cancers. Based on these signatures, they further classified the TME into four distinct subtypes that were conserved across 20 different cancer types (Bagaev et al. 2021). In contrast with the aforementioned classification methods, this approach considers not only immune cells but also cancer-associated fibroblasts (CAFs) (Bagaev et al. 2021). Indeed, analysis of biopsies from patients with pancreatic ductal adenocarcinoma suggested that CAFs constitute an essential basis for the classification of distinct TME subtypes (Grünwald et al. 2021). Therefore, the role of CAFs cannot be ignored when classifying TME types. However, owing to the heterogeneity and plasticity of CAFs (Luo et al. 2022), future classification based on CAFs should differentiate the different subtypes and functions of CAFs.

Krummel's team evaluated 364 unique tumors across different cancer types and subsequently discovered prominent patterns of immune composition across cancers via computational clustering of flow cytometry and transcriptome data (Combes et al. 2022). They established a classification system for TME subgroups with five major classes and twelve subclasses (namely, immune archetypes) that may be applied at the pan-cancer level. Each archetype is characterized by distinct combinations of cells and chemokines (Combes et al. 2023). Here, the role of chemokines in the TME and its classification is formally highlighted and clearly described. Distinct chemokine compositions can shape different tumor immune phenotypes and ultimately influence their responses to immunotherapy.

From certain cell types to specific cellular combinations, to the distinct biological characteristics of these cells, and ultimately to the precise chemokines that arrange these cellular assemblies, the growing understanding of the TME shows a continual deepening of expertise. We next investigate where chemokines guide the biological components of the microenvironment because they can lead cells to specific areas.

## 3. A roadmap for cell migration meticulously mapped by chemokines in the TME

### 3.1. Chemokines attract immune cells to intratumoral area

CXCR3 signaling is of critical importance for the homing of T cells within tumors and the efficacy of adoptive cell therapy (Mikucki et al. 2015). Patients who exhibited a high survival rate following immune checkpoint blockade (ICB) treatment demonstrated a positive correlation with high expression levels of CXCL9/10/11 and/or CXCR3, as determined by tumor tissue biopsies (Ayers et al. 2017; House et al. 2020). It has been established that the CXCL9/10/11-CXCR3 axis controls the migration, differentiation, and activation of immune cells, including type 1 T helper cells and CD8^+^ T cells (Tokunaga et al. 2018; He et al. 2024). Additionally, tumor tissue samples were obtained from 185 patients with high-grade serous ovarian cancer prior to chemotherapy, radiotherapy, or other anticancer treatments. The researchers discovered that CD8^+^ T cells were in close proximity to CXCL13 (Yang et al. 2021). The findings of preclinical studies demonstrated that CXCL13 facilitated the recruitment and activation of CD8^+^ T cells (Yang et al. 2021). Single-cell suspensions prepared from melanoma patient tumor samples were subjected to sorting CD4^+^ T cells, followed by single-cell targeted mRNA sequencing, which revealed CXCL13 expression in CD4^+^ T-cell populations (Veatch et al. 2022). In vitro studies showed that TGF-*β* promotes CXCL13 production by CD4^+^ T cells (Ukita et al. 2022). Overall, CD8^+^ T cells can be attracted to the tumor site by CXCL9/10/11/13, playing an important role in anti-tumor responses.

CXCR1/2, CX3CR1, and CCRL2 can regulate the homing of NK cells to tumor tissues (Lachota et al. 2023; Sozio et al. 2023). A randomized controlled clinical trial demonstrated that increasing the level of CXCL8 can promote the directional migration of NK cells positively, leading to prolonged overall survival (Walle et al. 2022). In the glioblastoma xenograft model, increased CXCL9 and CXCL10 expression was associated with enhanced NK-cell infiltration into tumors, suggesting that CXCL9 and CXCL10 may promote NK-cell recruitment to the TME (Shi et al. 2024).

CCL22 and CCL20 can attract T regulatory cell infiltration, which contributes to tumor immunosuppression (Wu et al. 2018; Röhrle et al. 2020; Mai et al. 2025). In vitro and in vivo studies have demonstrated that hypoxia induces HIF-1α-mediated CCL28 overexpression in HCC cells (Ren et al. 2016). This upregulation has been observed to attract Treg cells, which in turn amplifies Treg-driven angiogenesis, ultimately resulting in tumor progression (Facciabene et al. 2011; Ren et al. 2016). Similarly, using bioinformatic analyses together with in vitro and in vivo validation, Lu Ji and colleagues identified the *β*-catenin-CCL28-Treg cell axis as a key mechanism contributing to immunosuppression in the gastric TME (Ji et al. 2020).

The frequency of macrophage infiltration is positively correlated with CX3CL1 expression, according to a clinical multiscale immune profiling study that used removed tumors from patients who had not yet received treatment (Lavin et al. 2017). In vitro and in vivo experiments showed that CX3CL1 has the capacity to attract macrophages to the tumor site (Xiao et al. 2024). Furthermore, cancer cell-derived CX3CL1 can accelerate the recruitment of M2-polarized TAMs, thus contributing to the establishment of an immunosuppressive milieu (Huang et al. 2024). However, it's also possible that CX3CL1 helps attract CD8^+^ T cells to tumor locations, which would stop tumor growth in vivo (Zhang et al. 2022). Pancreatic ductal adenocarcinoma cells have the capacity to secrete the chemokines CCL2 and CCL7, which results in the recruitment of TAMs and myeloid-derived suppressor cells. This, in turn, leads to the development of immune resistance (Wan et al. 2023). Hypoxic cervical cancer cells stimulate CCL8 transcription, facilitating TAM infiltration via the CCR2-NF-κB pathway (Chen et al. 2019). In addition, the CCL2-CCR2 and CXCR4/CXCL12 axis have been shown to recruit macrophages, allowing tumor cells to elude the immune system and spread (Yang et al. 2020; Mezzapelle et al. 2022).

Furthermore, chemokines have the ability to draw neutrophils and DCs to the tumor. For instance, XCL1 expression is positively associated with tumor-infiltrating cDC1s and a better prognosis in individuals with melanoma (Kamei et al. 2024). The chemokine XCL1 plays a pivotal role in the recruitment of cDC1s, and its receptor XCR1 is expressed on cDC1s in a selective manner (Kamei et al. 2024). Immunohistochemical analysis of tumor tissues from 208 patients with lung cancer revealed that higher CXCL5 expression was associated with increased neutrophil infiltration and decreased CD8⁺ T-cell infiltration (Sun et al. 2024) (Figure 2).

**Figure 2.**
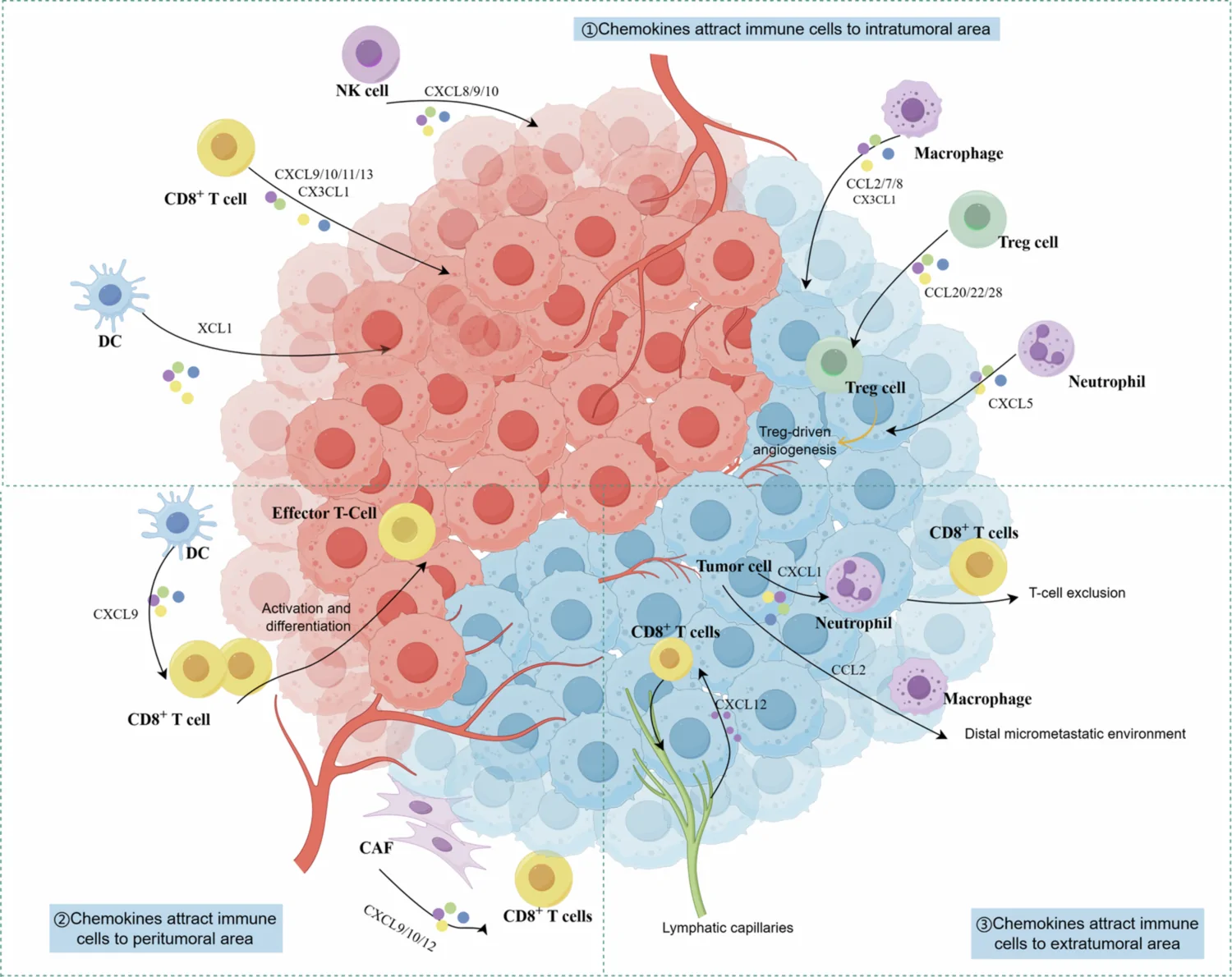
A roadmap for cell migration meticulously mapped by chemokines in the TME. Chemokines can not only attract immune cells into the interior and stromal areas of tumors, but also expel them out of the tumor. The red tumor area represents the role of chemokines in guiding immune cells to enhance the immune response. The blue tumor area represents the role of chemokines in guiding immune cells to exert immune suppression.

### 3.2. Chemokines attract immune cells to peritumoral area

Recent studies have demonstrated that TME enriched with CD90^+^CD44^+^ CAFs may be very resistant to ICB therapy, even when there is an abundance of T cells present (Dominguez et al. 2020; Kieffer et al. 2020; Bagaev et al. 2021). The spatial transcriptomes of distinct immunological types of head and neck squamous cell carcinoma revealed a CAF subset characterized by the secretion of CXCL9, CXCL10, and CXCL12, together with high expression of MHC class I molecules and galectin-9. These CAFs were associated with the retention of CD8⁺ T cells within the stroma and their limited infiltration into tumor nests (Arpinati et al. 2024; Li et al. 2024). Among fibroblast-derived chemokine signals, the CXCL12–CXCR4 axis represents a particularly well-characterized mechanism of T-cell spatial restriction. In pancreatic ductal adenocarcinoma (PDAC), activated pancreatic stellate cells and fibroblast activation protein-expressing CAFs constitute major stromal sources of CXCL12 (Feig et al. 2013). Through CXCR4-dependent signaling, CAF-derived CXCL12 recruits and retains T cells within stromal niches, spatially excluding them from tumor-cell regions and thereby limiting effective antitumor immunity (Feig et al. 2013). Accordingly, disruption of CXCL12–CXCR4 signaling can redistribute T cells toward tumor nests and enhance responses to immune checkpoint blockade in preclinical models (Ene-Obong et al. 2013). These findings highlight fibroblast-derived CXCL12 as an important spatial regulator of T-cell exclusion in the TME.

It should be noted that tumor stromal areas can act as a site for intratumoral T cell differentiation and activation. Philippa Meiser and colleagues discovered that MHCII^hi^CCR7^neg^ cDC1s can secrete the chemokine CXCL9, which attracts CD8 T cells to the stroma-tumor interface, thereby forming cDC1-CD8 T cell clusters that are preserved in human cancer (Meiser et al. 2023). In stromal niches, cDC1s activate TCF1CD8 T cells, resulting in the differentiation of effector T cells and enhanced anti-cancer immunity (Meiser et al. 2023).

### 3.3. Chemokines attract immune cells to extratumoral area

It is commonly known that chemokines can help tumor cells spread through lymphatic vessels (Karnezis et al. 2019; Deng et al. 2022). Interestingly, chemokines can also regulate the migration of T cells through lymphatic channels to leave tumors, hence impacting the efficacy of tumor immunotherapy (Figure 2). A study using the YUMMER1.7 mouse melanoma model found that CD8^+^ T lymphocytes at the tumor's periphery were adjacent to peritumoral lymphatic capillaries (Steele et al. 2023). Following more investigation into the mechanism, it was shown that tumor-associated lymphatic endothelial cells can release CXCL12, which causes CD8^+^ T cells to exit the tumor through lymphatic capillaries. This ultimately prevents CD8^+^ T cells from accumulating inside the tumor core (Steele et al. 2023).

CXCL1 has been found to be a key modulator of T-cell exclusion in human PDAC models that exhibit concomitant alterations in KRAS and TP53 (Bianchi et al. 2023). Specifically, it was found that CD8^+^ T cells are not present in cellular neighborhoods that include CXCL1^+^ PDAC-cells and CXCR2^+^ PMN-MDSCs. Mechanistically, the overexpression of CXCL1 in KRAS-TP53 co-altered human PDAC promotes contact between cancer cells and neutrophils, which in turn promotes feed-forward overproduction of CXCL1, ultimately resulting in the exclusion of CD8^+^ T cells (Bianchi et al. 2023).

In addition, chemokines secreted by tumor cells attract immune cells to other tissues or organs, creating a pro-metastatic microenvironment (Figure 2). For example, patients with colorectal cancer (CRC) frequently develop liver metastases, which may be attributed to the fact that the liver provides an environment conducive to the growth and proliferation of colorectal cancer cells (Wang et al. 2023). Macrophages were the most common cell type in the liver metastasis, according to single-cell analyses of autologous samples from individuals with colorectal cancer who had liver metastases (Zhang et al. 2019). Mechanically, CRC cells secrete CCL2, which attracts macrophages to the liver. These macrophages subsequently construct a favorable micrometastatic environment in the liver (Liu et al. 2022).

## 4. Factors affecting chemokine-guided immune cell migration

### 4.1. Immune cell interactions

Immune cells are important sources of chemokines. For example, NK cells, macrophages, and DCs can all produce the chemokines CXCL9 and CXCL10, which are induced by Interferon-*γ* (IFN-*γ*) (Spranger et al. 2017; Dangaj et al. 2019; Pascual-García et al. 2019; Meiser et al. 2023; Santana-Hernández et al. 2024). The recruitment of CD8^+^ T cells and the effectiveness of immune checkpoint blockage in melanoma brain metastases depend on NK cells, according to an in vivo study (Fife et al. 2024). Mechanistically, chemokines mediate the recruitment and infiltration of CD8^+^ T lymphocytes into brain melanoma metastases in both murine models and human tumor specimens. When NK cells are reduced, the intratumoral expression levels of chemokines that draw T cells drop significantly, which lowers T cell infiltration (Fife et al. 2024). Similarly, CD103^+^ DCs are required for CD8^+^ T cells recruitment, and their lack in malignancies leads to unsuccessful T cell recruitment, which is linked to the decreased expression of CXCL9 and CXCL10 from DCs (Spranger et al. 2017).

Meanwhile, some immune cells may have a negative impact on the efficient infiltration of tumor-fighting immune cells. Several cancer models have shown that CXCR3^+^ Treg cells preferentially connect with dendritic cells (DC1s) that produce CXCL9, hence inhibiting DC1s' priming of CD8^+^ T cells into tumors (Moreno Ayala et al. 2023). The depletion of Tregs leads to elevated levels of CXCL9 and CXCL10, resulting in enhanced T effector cell activity and their increased accumulation within the tumor (Akeus et al. 2018). As a result, the optimal immune cell combination will aid in the immunotherapeutic efficacy of tumors.

### 4.2. The expression levels of chemokine receptors on immune cells

The interaction between chemokines and their receptors on the surface of cells is a key mechanism regulating immune cell migration. Consequently, this migratory effect will be impacted by the reduction in the concentrations of these receptors on the immune cells. A study of CRC tissues from patients who had not received any therapeutic intervention revealed that the density of CD8^+^ T cells was lower in advanced-stage tumor tissues than in early-stage CRC tissues (Wang et al. 2020). From a mechanistic perspective, IL-17A levels were found to be elevated in sera from advanced-stage CRC patients. IL-17A can stimulate STAT3 signaling and then downregulate CXCR3 (the receptor of CXCL10) expression on CD8^+^ T cells, resulting in reduced CD8^+^ T cell migration (Wang et al. 2020).

Importantly, chemokine responsiveness is closely linked to the differentiation state of lymphocytes. Naïve T cells preferentially maintain lymphoid-homing programs characterized by CCR7-dependent responses to CCL19 and CCL21. This migratory program is largely retained by central memory T (T_cm_) cells but is substantially diminished in effector memory T (T_em_) cells (Sallusto et al. 1999; Weninger et al. 2001). Accordingly, naïve and memory T cells do not respond to completely distinct sets of chemokines; rather, differentiation progressively remodels their chemokine-receptor repertoire and migratory competence. Consistent with this concept, CCR7⁺ naïve and T_cm_ cells were preferentially enriched in malignant effusions, whereas the recruitment of CCR7^-^ effector-type and CCR5⁺ T cells was comparatively limited (Atanackovic et al. 2004). In metastatic melanoma, distinct patterns of CCR and CXCR expression across naïve, T_cm_, T_em_, and T_emra_ populations were associated with different patterns of metastatic dissemination and clinical outcomes (Jacquelot et al. 2016). Functionally, antigen-experienced CD8⁺ T cells display greater responsiveness to inflammatory chemokines than naïve cells (Weninger et al. 2001), and CXCL9 produced by XCR1⁺ dendritic cells can rapidly recruit CXCR3-expressing memory CD8⁺ T cells during recall responses (Alexandre et al. 2016). Differentiation-dependent remodeling of chemokine responsiveness is also evident in B cells; CXCR3 is absent from CD27^-^ naïve B cells but is expressed by a subset of memory B cells and can be further induced during their differentiation toward plasma-cell precursors (Muehlinghaus et al. 2005). Thus, the effects of chemokine networks depend not only on ligand availability but also on the differentiation-dependent expression and signaling competence of chemokine receptors on responding lymphocyte subsets.

### 4.3. Synergism between different chemokines

The cooperation of particular chemokines is essential for coordinating T-cell responses in tumor environments, as well as for promoting the development of T-cell inflammatory phenotypes and checkpoint immunotherapy responsiveness. According to Dangaj et al., for instance, T cell infiltration into tumors depends on CCL5 expressed by tumor cells and CXCL9 expressed by macrophages and DCs (Dangaj et al. 2019). In particular, CCL5 from tumor cells stimulates T-cell engraftment in solid tumors by driving CXCL9 production in TAMs via IFNγ. Tumor immune desertification in vivo can be explained by an epigenetic decrease of CCL5 (Dangaj et al. 2019).

### 4.4. Treatment regimens

A study in vivo using a mouse model of breast cancer demonstrated that the expression levels of CXCL9 and CXCL10 were considerably increased after combined PD-1/CTLA-4 inhibition (House et al. 2020). Intratumoral production of CXCL9 and CXCL10 is primarily induced by IFN-*γ* in macrophages subsequent to dual checkpoint inhibition. Meanwhile, combined PD-1/CTLA-4 suppression enhances intratumoral CD8^+^ T cell frequencies in a CXCR3-dependent manner. In vivo and in vitro studies have shown that tumor cell PD-L1 engagement with macrophage PD-1 can promote SHP2 signaling in macrophages and consequently decrease type I interferon. The lack of type I interferon signaling in macrophages decreases CXCL9 and CXCL10 production, suppressing cytotoxic T lymphocytes' tumor infiltration and driving metastasis from the primary tumor (Klement et al. 2023). Interestingly, when PD-1-SHP-2 signaling is blocked, myeloid cells in tumor-bearing mice can exhibit enhanced differentiation, activation, phagocytosis, and inflammatory responses (Christofides et al. 2023). This may result in reduced tumor growth (Christofides et al. 2023). Taken together, allosteric inhibitors of SHP-2 are a promising class of tumor immunotherapeutic agents that promote T-cell infiltration and enhance macrophage immune responses.

Human epidermal growth factor receptor 2 (HER2) positive breast cancer patients receiving anti-HER2 antibody-based neoadjuvant treatment showed strong correlations between the number of tumor-infiltrating NK cells and the production of CCL5, CXCL9, and CXCL10 (Santana-Hernández et al. 2024). In vitro and in vivo mechanistic studies have demonstrated that the systemic administration of anti-HER2 antibodies can activate tumor-infiltrating NK cells, leading to the production of CCL5/IFN-*γ*. This, in turn, stimulates the production of CXCL9/10 from bystander breast cancer cells (Santana-Hernández et al. 2024). Furthermore, elevated serum levels of CCL5 and CXCL9 were observed in patients who exhibited a good response to three cycles of immunotherapy with anti-HER2 antibody-based neoadjuvant treatment (Santana-Hernández et al. 2024). Consequently, CCL5 and CXCL9 may serve as potentially useful biomarkers of response to anti-HER2 antibody therapy.

Tumor immune deserts and immunotherapy resistance can be reversed with low-dose radiation (Zhai et al. 2022). The findings of a randomized controlled clinical study indicated that low-dose radiation therapy enhances the infiltration of T cells into tumors (Timke et al. 2011). Mechanistically, local low-dose gamma irradiation can polarize macrophages into the M1 type. Following this, M1 macrophages produce the necessary chemokines to draw in effector T cells. They also trigger an inflammatory response and help the tumor vasculature return to normal, which makes the environment conducive to T-cell infiltration (Klug et al. 2013). Similarly, low-dose irradiation can induce immune cell infiltration in gastric cancer and ovarian cancer (Herrera et al. 2022; Zhou et al. 2024). However, low-dose radiation combined with durvalumab and tremelimumab did not increase the overall response rate in patients with metastatic non-small-cell lung cancer who had not responded to previous PD-L1 therapy (Schoenfeld et al. 2022). Thus, the reversal of immune-desert tumors by low-dose radiotherapy is a complex process that is not as straightforward as previously assumed.

## 5. Nanodelivery systems integrated with chemokines and chemokine receptors

### 5.1. Nanoparticles modified with chemokines or their receptors

Compared with conventional therapeutic strategies, nanoparticles can achieve more selective drug accumulation at tumor sites via the enhanced permeability and retention (EPR) effect, surface ligand functionalization, and controlled drug release, thereby improving therapeutic efficacy while reducing systemic toxicity. Recent advances in TME-responsive nanomedicine have further demonstrated that stimulus-responsive nanocarriers can enable site-specific drug release and diagnostic imaging while minimizing systemic toxicity (Guo et al. 2025). Nanoparticle systems can be broadly classified into three categories: inorganic nanoparticles (e.g. magnetic and mesoporous silica nanoparticles), organic nanoparticles (e.g. PLGA nanoparticles), and hybrid nanoparticle platforms (Ma et al. 2025). As drug delivery vehicles, nanoparticles carry chemokines to the tumor site, where they are continuously released to draw in the required immune cells. Chemokines typically include four conserved cysteine residues at conserved locations. These cysteines form disulfide connections that support chemokines' tertiary structure. The kind of chemokine is determined by the distance between the first two cysteine residues (Miller and Mayo 2017; Kleist et al. 2025). The majority of chemokines are known to be positively charged (Messina et al. 2024). Electrostatic interactions serve as a key mechanism mediating the binding of chemokines to nanoparticles. The surface charge of nanoparticles mainly depends on the type of material (Zheng et al. 2024) (Table 1 and Figure 3).

**Figure 3.**
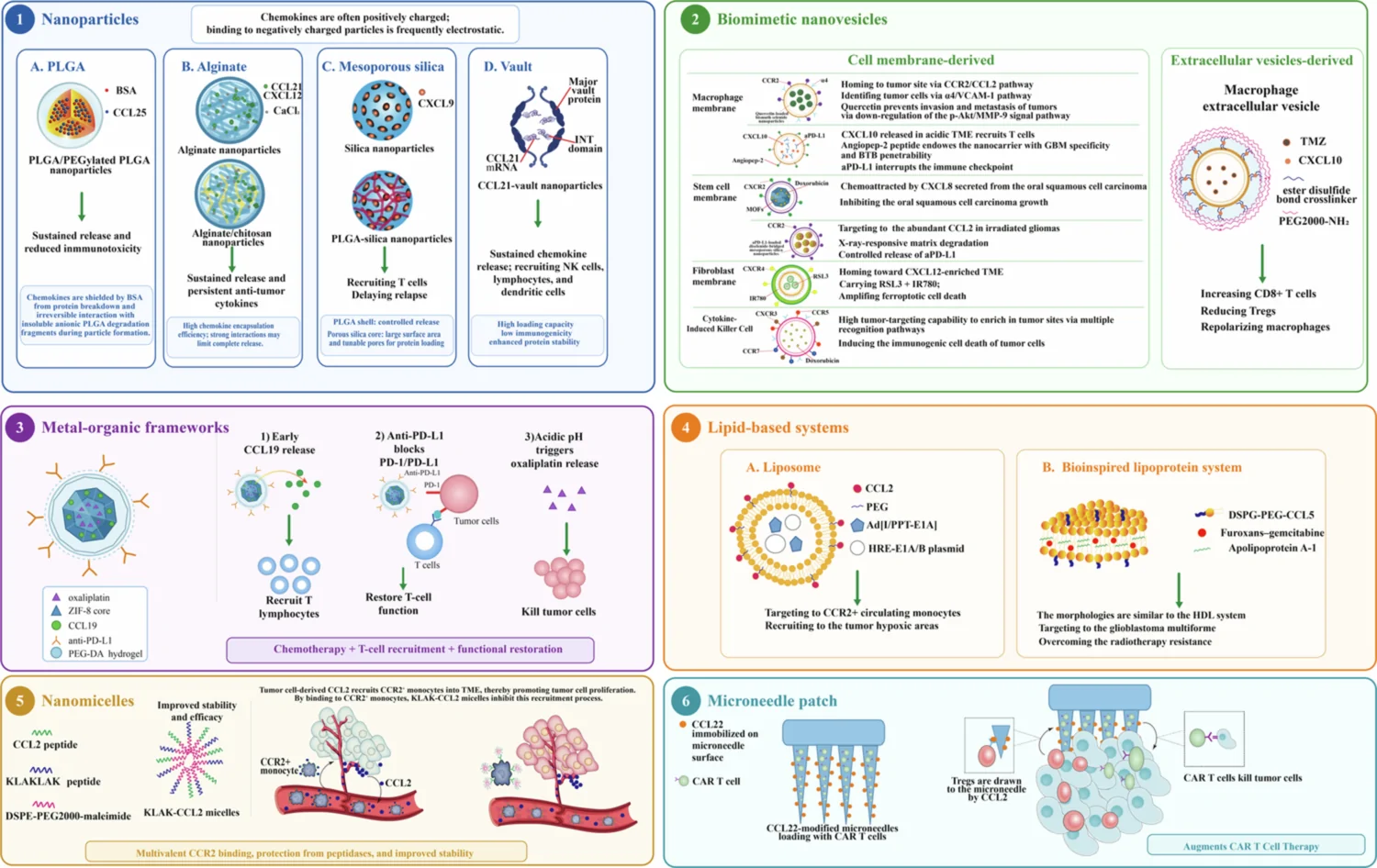
Chemokine- and chemokine receptor-integrated nanodelivery systems for cancer therapy. The primary goal of a variety of nanomaterials, such as polymeric nanoparticles, alginate-based particles, mesoporous silica systems, vault particles, MOFs, lipid-based carriers, nanomicelles, extracellular vesicles, and microneedle patches, is to enhance the biocompatibility, stability, and sustained release of chemokines while shielding protein bioactivity from deterioration or damage related to fabrication. These tactics aid in creating local chemokine gradients and controlling the recruitment of immune cells in the tumor microenvironment. On the other hand, chemokine receptor-decorated nanocarriers or receptorderived targeting modules mainly take use of tumor-associated chemokine gradients to improve anticancer drug delivery and tumor homing. These platforms work together to coordinate tumor targeting, immune regulation, and therapeutic delivery via chemokine biology.

**Table 1.** Non-biomimetic nanocarriers integrated with chemokines or chemokine receptors.

Modified chemokine/receptor	Targeted chemokine/receptor	Drug deliver system	Main preparing method	Functions	Ref.
CCL21	Not mentioned	CCL21- loaded alginate nanoparticles	Ionotropic gelation processes	Inhibiting tumor growth; increasing the level of anti-tumor cytokines; Increasing the number of immune cells with anti-tumor activity	Miller and Mayo (2017)
CXCL12	Not mentioned	Alginate/chitosan nanoparticles entrapping CXCL12	Ionotropic gelation method	Reversing the migration direction of glioblastoma multiforme cells	Kleist et al. (2025)
CCL21	Not mentioned	CCL21-packaged vault nanoparticles	Baculovirus expression system	Recruiting lymphocytes and dendritic cells; enhancing host antitumor response	Poelaert et al. 2020; Ruiz-Hitzky et al. (2026)
CCL20	Not mentioned	PLGA-PEG nanoparticles loaded with a NIR-dye for theranostic purposes, doxorubicin cytostatic agent, poly (I:C) and R848 immune adjuvants, and CCL20 chemokine	Solvent evaporation-extraction method	Recruiting more immune cells into the tumor; promoting tumor cell death	Guo et al. (2025)
CXCR4	Not mentioned	CXCR4-PEG-CdTe-DOX: polyethylene glycol-modified cadmium telluride	Chemical coupling; electrostatic interaction	improving the antitumor activity of DOX	Ghezzi et al. 2021; Chen et al. (2024)
CXCL9	CXCR3	CXCL9 was encapsulated in microparticles composed of a silica core and a PLGA outer shell	Chemical synthesis	attracting CD8+ T cells to tumor	Fröhlich et al. (2018)
CCL25	CCR9	CCL25-loaded PLGA particles	the water-in-oil-in-water double emulsion;solvent evaporation	recruiting and activating macrophages and T cells; inducing cytokine secretion	Schoenfeld et al. 2022; Zhou et al. (2024)
CCL19	CCR7	A metal-organic framework nanosystem armed with PD-L1 antibody and co-encapsulating CCL19 and oxaliplatin	continuous stirring	Recruiting more T cells	Wang et al. (2026)
CCL5	Not mentioned	C‐LNG: CCL5 modified, bioinspired lipoprotein nanoparticles loaded with a reduction-sensitive nitric oxide donor–gemcitabine conjugate	Chemical coupling	Targeting glioblastoma multiforme; overcoming RT resistance	Liu et al. (2022)
CCL2	CCR2	CCL2-coated liposomes encapsulating an oncolytic adenovirus	film hydration method	Killing prostate cancer cells; targeting circulating monocytes	Fang et al. (2023)
CCL2	CCR2	CCL2-micelle	thin-film hydration; Chemical synthesis	reducing tumor growth and TAM infiltration	Chaudhari et al. (2026)
CCL22	Not mentioned	A CCL22-functionalized grooved microneedle patch	ice-templated method	Augmenting adoptive T cell therapy; remodeling of the tumor immune microenvironment by redirecting Treg cells and increasing the Teff/Treg ratio	Abánades Lázaro et al. (2024)

#### 5.1.1. PLGA nanoparticles

One well-known example of negatively charged nanoparticles is poly (lactic-co-glycolic acid) (PLGA) nanoparticles. Kristin Fröhlich et al. used a water-in-oil-in-water (w1/o/w2) double-emulsion solvent evaporation process to produce CCL25-loaded PLGA nanoparticles, with CCL25 absorbed into the internal aqueous phase (Fröhlich et al. 2018). Bioactive CCL25 can be released more effectively by BSA-containing PLGA particles (Fröhlich et al. 2018). Because BSA has a net negative charge at physiological pH, it can interact electrostatically with positively charged areas of CCL25 (Böhme and Scheler 2007). These connections may decrease the chemokine's vulnerability to acid-mediated loss of function and protect them from irreversible affiliation with insoluble anionic PLGA breakdown fragments. Furthermore, by minimizing CCL25's direct contact with organic solvents, decreasing interfacial adsorption, and inhibiting protein aggregation, BSA can serve as a protective carrier protein during particle production. When taken as a whole, these actions enhance chemokine stability in the PLGA matrix and promote a higher recovery of functionally active CCL25 upon release (Fröhlich et al. 2018). The subsequent CCL25 immunotoxicity report explains why controlled release is essential rather than just practical. Peripheral blood mononuclear cells (PBMCs) and immunological subsets were activated by direct high-dose CCL25 stimulation, which also increases monocyte chemoattractant protein-1 (MCP-1), interleukin-8 (IL-8), interleukin-1β (IL-1β), tumor-necrosis-factor-*α* (TNF-*α*), and IFN-*γ*, upregulates human leukocyte antigen-DR (HLA-DR), and encourages leukocyte movement. Supernatants from CCL25-loaded PLGA microparticles, on the other hand, did not exhibit significant surface-marker activation or migration and only moderately increased MCP-1, IL-8, and IL-1β (Spinnen et al. 2021). Furthermore, Da Silva et al. developed biodegradable nanoparticles for the co-delivery of doxorubicin and immune adjuvants and demonstrated their therapeutic efficacy through in vitro and in vivo studies in treatment-resistant solid tumor models (Da Silva et al. 2019). This formulation combines many therapeutic actions into a single carrier: poly(I:C)/R848 activates innate immune signaling and dendritic-cell maturation, CCL20 attracts CCR6-expressing immune cells to the tumor, and doxorubicin directly kills tumor cells and stimulates tumor antigen release. It should be mentioned, though, that by day 12, chemokine release in this multi-drug PLGA nanoparticle system had already dropped below the detection limit (Da Silva et al. 2019). On the other hand, chemokine-only PLGA nanoparticles were able to maintain measurable chemokine release for up to 30 days (Fröhlich et al. 2018). The possible impact of co-loaded medications on chemokine encapsulation stability, release behavior, and bioactive recovery should therefore be carefully taken into account while developing delivery systems for chemokines.

#### 5.1.2. Alginate nanoparticles

Because alginate is negatively charged, easy to handle, biocompatible, and capable of interacting electrostatically with positively charged proteins, alginate-based systems are particularly appealing for chemokines (Ruiz-Hitzky et al. 2026). CCL21-loaded alginate nanoparticles can be created utilizing an ionotropic gelation method. To create a pre-gelled alginate network, the alginate solution is combined with CCL21 and Pluronic F127 before gradually adding CaCl₂ (Poelaert et al. 2020). Pluronic F127 strengthens the nanoparticle matrix and aids in the sustained release of the encapsulated protein. Protamine sulfate is then added to induce polyelectrolyte complexation with alginate, stabilizing the pre-gel nuclei and aiding in the production of distinct nanoparticles (Poelaert et al. 2020). In vivo, nanoformulated CCL21 was more effective than free CCL21 at slowing tumor growth and increasing survival; a fraction of treated animals experienced complete tumor clearance with rechallenge protection. Nanoformulated CCL21 increases the persistence of anti-tumor cytokines such as CXCL9, CXCL10, IFN-*γ*, IL-12p70, CCL3, and CCL2, while lowering IL-6 and IL-16 levels compared to free CCL21 (Poelaert et al. 2020). CXCL12-loaded alginate/chitosan nanoparticles exhibited high encapsulation efficiency, while in vitro studies confirmed that they were non-cytotoxic, did not promote F98 glioblastoma cell proliferation, and preserved the chemotactic activity of released CXCL12 (Gascon et al. 2020). Mathematical modeling suggested diffusion-dominated release with strong CXCL12-alginate interactions (Gascon et al. 2020). Alginate and chemokines have significant electrostatic interactions, which prevent the payload from being fully released from the nanoparticles. The greatest recorded cumulative release was about 60%, and it rises with the original loading amount (Gascon et al. 2020). A significant question is raised by this observation: is it possible that the leftover chemokine will be released in a secondary burst when the alginate-based substance ultimately breaks down? It is unknown and needs more research if such an abrupt local rise in the chemokine concentration within the tumor site will trigger extra or unwanted immune responses.

#### 5.1.3. Mesoporous silica nanoparticles

Mesoporous silica nanoparticles (MSNs) are an excellent drug carrier due to their huge surface area, high volume ratio, superior biocompatibility, and controlled surface functionalization (Lérida-Viso et al. 2023; Guo et al. 2025). PLGA-silica microparticles were created by Gabriele Romano et al. for the delivery of rCXCL9 (Romano et al. 2023). The PLGA shell regulates release, while the porous core offers a large surface area and adjustable pores for protein loading. The rCXCL9 encapsulation efficiency was approximately 39.5%, and the silica particles were approximately 371 nm before coating and 1003 nm following PLGA encapsulation. In vitro experiments showed that the PLGA microparticles efficiently released rCxcl9 and promoted mouse CD8^+^ T-cell migration, with 10% PLGA inducing the highest migratory response and therefore being selected for subsequent in vivo studies. In vivo melanoma models further confirmed the CXCL9-mediated recruitment of CD8^+^CXCR3^+^ T cells. In combination with BRAFi, rCXCL9-loaded PLGA microparticles significantly delayed tumor relapse. Moreover, adding rCXCL9-loaded PLGA microparticles to standard combined BRAFi and MEKi treatment further improved therapeutic efficacy and induced complete pathological responses in most tumors (Romano et al. 2023). However, formulation complexity is the biggest disadvantage. Translation would necessitate careful control of porous-core size, pore design, PLGA thickness, release repeatability, and long-term local safety. The encapsulation efficiency is lower than that of alginate systems, and the size approaches the micron scale after coating.

#### 5.1.4. Vault nanoparticles

Vault nanoparticles are naturally occurring nanocapsules that resemble ribonucleoproteins and are designed to contain medicinal payloads (Buehler et al. 2014; Ding et al. 2018). Similar to endogenous vaults, engineered vaults may hold hundreds to thousands of low-molecular-weight molecules and are non-immunogenic (Buehler et al. 2011). The primary component of vault nanoparticles, which are hollow protein nanocapsules, is the major vault protein (MVP). The INT domain of a vault-associated protein called VPARP can attach to the inner surface of MVP, enabling the loading of INT-fused proteins into the vault lumen. CCL21 was genetically fused to the INT domain, and the resulting CCL21-INT fusion protein was combined with recombinant empty vaults to create CCL21-vault nanocapsules. CCL21 was integrated into the vault interior by INT-mediated contact with MVP, offering a shielding nanocapsule for chemokine distribution (Kar et al. 2011). Compared to free CCL21, empty vaults, or PBS, CCL21-packaged vault nanoparticles decreased the tumor volume more successfully in an in vivo model of glioma, and the CCL21-vault group was the only one whose average tumor volume changed by a negative percentage (Voth et al. 2020). Vault nanoparticles enhanced the gradient-dependent release and stability of CCL21, which may promote the recruitment of NK cells, lymphocytes, and dendritic cells. The fact that this evidence is still preliminary is a drawback. The long-term survival benefit, biodistribution, manufacturing scalability, and precise release kinetics were not fully established by the current study. Fragile chemokines may find vaults appealing, but their manufacturing, repeatability, and regulatory routes are less developed than those of PLGA or alginate.

### 5.2. Biomimetic nanovesicles modified with chemokines or their receptors

By maintaining membrane proteins, adhesion molecules, immune-regulatory ligands, and chemokine receptors from source cells, biomimetic nanovesicles offer a physiologically active interface for drug administration (Herrmann et al. 2021; Graván et al. 2025). In contrast to traditional synthetic nanoparticles, these systems can enhance tumor homing, immune-cell recruitment, and local immune activation by taking advantage of endogenous chemotactic pathways, such as CCL2/CCR2, CXCL8/CXCR2, CXCL12/CXCR4, and CXCL10/CXCR3 (Fang et al. 2023). Biomimetic nanovesicles can generally be made in two complementary ways: either by transporting chemokines inside or on the vesicle surface to change the immunological environment, or by showing particular chemokine receptors on their surface to follow chemokine gradients. More research has been done on the former strategy (Table 2).

**Table 2.** Biomimetic membrane-coated nanocarriers integrated with chemokines or chemokine receptors.

Modified chemokine/receptor	Targeted chemokine/receptor	Cellular platform	Drug deliver system	Main preparing method	Functions	Ref
CXCR4	CXCL12	Murine dermal fibroblasts	FbM@IR/RSL3 NPs: FbM shell (CXCR4-overexpressing fibroblast membrane)+IR/RSL3 NP core (IR780-conjugated lipid nanoparticle encapsulating ferroptosis inducer RSL3	Lentiviral transfection; solvent diffusion method; sonication	Preferential tumor accumulation in multiple preclinical models exhibiting high CXCL12 secretion	(Graván et al. 2025)
CXCR3, CCR5 and CCR7	CXCL9, CCL5 and CCL21	Cytokine-induced killer cells	DOX@CpG-PNVs: PD-1-engineered CIK cell-derived nanovesicles enriched in tumor chemokine receptors, and loaded with DOX plus CpG ODN	Splenic mononuclear cells treated with IFN-*γ* and IL-2; physical extrusion	Superior tumor-targeting ability	(Zhao et al. 2018)
CXCL10	Not mentioned	Macrophage-derived extracellular vesicles	TC@MEVs: CXCL10-conjugated, PEGylated TMZ-loaded M-EV membrane-derived vesicles	Ultra-high-speed centrifugation; physical extrusion	Temporary disruption of the blood-brain barrier; recruiting CD8+ T cells; killing tumor cells	(Xu et al. 2023)
CXCL10	CXCR3	Murine macrophages	A2-MPC nanovesicles: Angiopep-2-decorated, macrophage membrane-camouflaged nanovesicles co-loaded with CXCL10 and anti-PD-L1	Sonication;freeze-thawing cycles;extrusion	Targeted delivery to brain tumor regions;recruiting CD8+ T cells;	(Buehler et al. 2014)
CCR2	CCL2	Mesenchymal stem cell	CCR2-SCM@MSN@αPD-L1: CCR2-overexpressing MSC membrane-camouflaged, αPD-L1-loaded diselenide-bridged mesoporous silica nanoparticles	Extrusion	Enhanced and specific GBM radioimmunotherapy via radiation-guided tumor accumulation, X-ray-responsive αPD-L1 release, and selenium-mediated radiosensitization	(Buehler et al. 2011)
CXCR2	CXCL8	Dental pulp mesenchymal stem cell	MOF@DPSCM: CXCR2-bearing dental pulp mesenchymal stem cell membrane-coated, DOX-loaded metal-organic framework nanoparticles	Solvent method	Blocking oral squamous cell carcinoma growth effectively	(Ding et al. 2018)
CCR2	CCL2	Murine macrophages	M@BS-QE NPs:Macrophage membrane-camouflaged hollow bismuth selenide nanoparticles loaded with quercetin	Hydrothermal process method	Improved tumor cells responsiveness to photothermal therapy	(Romano et al. 2023)
CXCR4, CCR2	Not mentioned	Human umbilical cord mesenchymal stem cells	Human umbilical cord mesenchymal stem cells membrane loaded with mitoxantrone	Cryo-treating; co-incubation	selectively accumulating in tumors	(Rahnama et al. 2024)

#### 5.2.1. Cell membrane-derived biomimetic platforms

Macrophage membranes are one of the most popular cellular platforms for chemokine-axis-guided administration. For example, M@BS-QE NPs are hollow bismuth selenide nanoparticles loaded with quercetin that mimic the membrane of macrophages (Zhao et al. 2018). The macrophage membrane (M) functions as the outer shell in this formulation, conferring extended circulation, CCL2/CCR2-mediated tumor recruitment, α4 integrin/VCAM-1-dependent active targeting, and immune evasion. In vitro and in vivo studies demonstrated that these properties contributed to enhanced tumor accumulation of macrophage-camouflaged NPs. Quercetin (QE) loaded in the inner core can be released upon near-infrared (NIR) irradiation. In vivo studies demonstrated that M@BS-QE NPs effectively suppressed breast cancer lung metastasis by downregulating heat shock protein 70 (HSP70) and inhibiting the *p*-Akt/MMP-9 signaling pathway (Zhao et al. 2018). In order to create M@BS-QE nanoparticles, hollow mesoporous Bi_2_Se_3_ nanoparticles were first synthesized and then loaded with QE to create BS-QE cores. Low-temperature sonication, sucrose-assisted homogenate processing, differential centrifugation, and washing in ice-cold Tris-magnesium buffer were used to separate the macrophage membranes from RAW 264.7 cells. In order to create macrophage membrane-camouflaged M@BS-QE nanoparticles, the purified macrophage membrane vesicles were combined with BS-QE nanoparticles and repeatedly extruded through 400 nm polycarbonate membranes (Zhao et al. 2018). Additionally, macrophage membranes can be employed as biomimetic carriers to directly transport chemokines. In the macrophage membrane-derived nanovesicles denoted as A2-MPC, CXCL10, and an anti-PD-L1 antibody are co-encapsulated through a sonication-freeze-thaw-extrusion process (Xu et al. 2023). Angiopep-2 surface modification is then applied to improve the blood–tumor barrier in penetration. In the acidic tumor environment, CXCL10 is released to attract T lymphocytes, increasing the effectiveness of PD-L1 inhibition. In a glioma-bearing mouse model, intravenously administered A2-MPC exhibited enhanced blood‒brain barrier penetration and superior therapeutic efficacy (Xu et al. 2023). These findings suggest that delivering chemokines to remodel the immunosuppressive microenvironment of ‘cold’ tumors and thereby broaden the applicability of immune checkpoint inhibitors represents a promising therapeutic strategy.

Zhou et al. developed dental pulp stem cell membrane‑coated metal–organic framework nanoparticles for doxorubicin delivery, which effectively suppressed oral squamous cell carcinoma growth both in vitro and in vivo (Zhou et al. 2021). The targeting capability of this approach was mostly dependent on the enrichment of CXCL8/IL-8 in the tumor microenvironment and the natural or tumor-conditioned expression of CXCR2 on the stem cell membrane. The stem cell membrane serves as a biological interface for tumor homing, while the MOF core offers drug-loading capacity and pH-responsive release (Zhou et al. 2021). This design exemplifies a traditional ‘biomimetic membrane plus engineered nanocore’ approach, wherein the interior nanomaterial controls payload capacity and release behavior while the membrane provides biological recognition. A more sophisticated method involves engineering MSCs prior to membrane extraction, which allows the final nanocarrier to inherit a predetermined chemokine receptor phenotype. In a recent glioblastoma investigation, CCR2-overexpressing MSC membranes were coated on diselenide-bridged mesoporous silica nanoparticles containing anti-PD-L1 antibodies (Wang et al. 2024). The reason for this design stemmed from the fact that radiation elevated CCL2 levels in the glioblastoma microenvironment. Overexpressing CCR2 in MSCs prior to membrane isolation allowed the resulting CCR2-engineered membrane-coated nanoparticles to respond to the CCL2 gradient and achieve increased lesion-specific accumulation. Compared to natural MSC membrane coating, this technique is more rational and controllable because the chemokine axis is specifically tailored to the disease microenvironment. Furthermore, the interior nanocore was more than just a passive drug reservoir; the diselenide-bridged mesoporous silica responds to X-ray/ROS activation, releasing anti-PD-L1, and promoting radiosensitization via selenium-mediated actions (Wang et al. 2024).

A safer and more scalable substitute for tumor-cell or immune-cell membranes is fibroblast membranes (Sun et al. 2022). Dermal fibroblasts are extensible, clinically accessible, and less prone to spreading carcinogenic substances (Rahnama et al. 2024). However, a chemokine-navigation module can be installed using receptor engineering since native fibroblast membranes might not have enough tumor homing. In a recent example, CXCR4-engineered fibroblast membrane nanovesicles were employed to drive tumor homing using CXCL12 (Bai et al. 2025). After dermal fibroblasts were genetically altered to overexpress CXCR4, their membrane vesicles were separated and applied to lipid nanoparticles that had been co-loaded with RSL3 and IR780. While IR780 allows near-infrared photothermal heating and RSL3-induced ferroptosis by blocking GPX4, the CXCR4-overexpressing membrane guided the nanoparticles toward CXCL12-enriched tumor microenvironments.

Additionally, because cytokine-induced killer cells have traits with both T cells and natural killer cells, they have attracted special study. They might express a variety of chemokine receptors and ligands that might cause apoptosis (Ying Li et al. 2024; Rettinger et al. 2026). IFN-*γ*, an anti-CD3 antibody, and IL-2 were used to induce CIK cells from splenic mononuclear cells in the designed cytokine-induced killer (CIK) cell-derived nanovesicle system (Wang et al. 2026). These cells were subsequently genetically altered to overexpress PD-1. To create PD-1-expressing nanovesicles, PD-1-expressing CIK cells were extruded through membranes with progressively smaller pores. These vesicles contain apoptosis-inducing ligands such as FasL and TRAIL, as well as tumor chemokine receptors such as CXCR3, CCR5, and CCR7. To create DOX@CpG-PNVs, doxorubicin was injected into the nanovesicles, and CpG oligonucleotide was affixed to the vesicle surface. This method combines doxorubicin-induced immunogenic cell death, PD-1/PD-L1 inhibition, chemokine receptor-mediated tumor homing, direct tumor killing, and CpG-mediated innate immune activation. Even in a tumor model with low immunogenicity, it produced potent anticancer effects.

#### 5.2.2. Extracellular vesicle-derived biomimetic platforms

TC@MEVs are based on naturally occurring extracellular vesicles (EVs) generated from macrophages (Dong et al. 2025). Temozolomide (TMZ) is loaded into the vesicular structure by sonication and extrusion, and CXCL10 is coupled onto the vesicle surface via a redox-cleavable disulfide linker. TC@MEVs release CXCL10 and TMZ in glioblastoma under ultrasound-mediated blood‒brain barrier opening and high-glutathione circumstances, which improves chemo-immunotherapy, increases CD8⁺ T-cell infiltration, decreases Tregs, and repolarizes macrophages (Dong et al. 2025).

EVs and cell membrane-derived carriers both have good biocompatibility and comparatively low immunogenicity, but each platform has various drawbacks (Liew et al. 2024). The capacity of cell membrane-derived carriers to partially inherit the inherent chemotactic and tissue-homing characteristics of their parent cells is a significant benefit, allowing for more effective lesion-targeted delivery (Fang et al. 2023). However, traditional mechanical preparation techniques such as extrusion may result in the loss, inactivation, or aberrant orientation of functional membrane proteins, especially adhesion molecules and chemokine receptors, which would ultimately impair the membrane-based carriers' capacity to target chemotactics (Liu et al. 2022). Wang et al. described a cryo-trojan mesenchymal stem cell platform and demonstrated that cryo-treatment eliminated proliferative risks while preserving chemokine receptors and cellular structural integrity, therefore preserving MSC-derived tumor-homing capacity (Wang et al. 2025). This discovery implies that cryo-based processing techniques could be a viable path for improving the creation of biomimetic delivery systems derived from cells or cell membranes. EVs, on the other hand, have great potential as natural delivery vehicles and are significant endogenous mediators of intercellular communication (Chaudhari et al. 2026). However, low yields and significant heterogeneity continue to limit EV-based treatments, which provide significant obstacles to scalable production, quality assurance, and batch-to-batch consistency (Du et al. 2023).

### 5.3. Metal-organic framework encapsulating chemokines

Because of their porous structure, adjustable coordination chemistry, and modifiable surface, which enables the integration of therapeutic proteins, small-molecule medications, and immune-modulating ligands within a single nanosystem, metal-organic frameworks (MOFs) are emerging as highly programmable nanocarriers for chemokine delivery (Abánades Lázaro et al. 2024). A typical MOF-based chemokine delivery system for osteosarcoma immunochemotherapy was described by Li et al. (2025). The formulation, known as OC@ZPP nanoparticles, was made up of surface-conjugated anti-PD-L1, a PEG-DA layer containing CCL19, and a ZIF-8 core filled with oxaliplatin. The spatial isolation and sequential operation of several therapeutic modules were made possible by this hierarchical design. In addition to blocking the PD-1/PD-L1 immune checkpoint axis, anti-PD-L1 on the nanoparticle surface enhanced the identification of PD-L1-positive tumor cells. While oxaliplatin was stored in the ZIF-8 core and released subsequently in reaction to acidic pH following tumor-cell internalization, CCL19, which was integrated within the outside PEG-DA layer, was intended for early release to attract T lymphocytes into the tumor microenvironment (Li et al. 2025). The integrated design enables both high drug-loading capacity and programmed drug release, demonstrating marked antitumor effects across in vitro and in vivo settings.

Although both MOFs and mesoporous silica nanoparticles are porous nanocarriers, their drug-delivery mechanisms are different. Within the organized mesoporous channels of MSNs, drug loading mostly depends on physical adsorption, electrostatic interactions, hydrogen bonding, or hydrophobic interactions (Xu et al. 2023). They are especially well-suited for loading small-molecule chemotherapeutics, hydrophobic medicines, photosensitizers, siRNAs, and proteins due to their well-defined pore geometries, high specific surface area, and easily changeable surface silanol groups. Then, ‘pore-gating’ techniques employing polymers, lipids, peptides, disulfide bonds, or pH-sensitive caps can be used to accomplish responsive release (Xu et al. 2023). Drug loading in MOFs can take use of coordination interactions between metal nodes and functional groups in pharmaceuticals, such as carboxyl, phosphate, amino groups, or heterocycles containing nitrogen, in addition to pore adsorption (Abánades Lázaro et al. 2024). Moreover, ‘one-pot’ coprecipitation, in situ mineralization, or metal–ligand self-assembly can be used to directly insert medications or proteins into the framework. Because of these characteristics, MOFs have a special ability to transport biomacromolecules such as proteins, nucleic acids, enzymes, and chemokines. MOFs offer more chemical tunability but necessitate a more thorough assessment of metal-ion release, framework degradation, repeatability, and long-term biosafety.

### 5.4. Lipid-based chemokine-functionalized delivery systems

With several approved formulations and a considerable body of clinical evidence, liposomes are among the first nanodrug delivery methods to be successfully translated into clinical settings (Filipczak et al. 2020). Recent research has further combined chemokines with liposomal delivery systems. Chemokines can be added to liposomal systems as surface-conjugated ligands, soluble proteins in the aqueous core, or lipid-anchored chemokine-mimetic peptides. CCL2-coated liposomes containing an oncolytic adenovirus and a hypoxia-responsive plasmid were reported by Iscaro et al. (2022). This system reduces tumor growth and pulmonary metastasis while requiring approximately 1000 times less virus than free adenovirus. Surface CCL2 was used to target circulating monocytes that expressed CCR2, which then served as cellular ‘Trojan horses’ to transport the viral payload into hypoxic tumor regions. In this method, circulating monocytes that expressed CCR2 were targeted by surface CCL2, which subsequently served as cellular ‘Trojan horses’ to carry the viral payload into hypoxic tumor areas. This approach used around 1000 times less virus than free adenovirus while reducing tumor development and lung metastasis (Iscaro et al. 2022). For glioblastoma radiation sensitization, Sun et al. created a bioinspired lipoprotein system modified with a CCL5-mimetic peptide (Sun et al. 2024). The formulation employed a DSPE-PEG-CCL5 module to improve tumor targeting and integrated a furoxan–gemcitabine combination into an HDL-like lipid nanostructure. This method overcame radiotherapy resistance by preferentially accumulating in orthotopic glioblastoma, responsively releasing gemcitabine and nitric oxide under reductive circumstances, and producing peroxynitrite during X-ray irradiation. These studies show that liposomes can leverage chemokine biology to design immune-cell hitching, tumor targeting, and combination therapy in addition to delivering chemokines (Sun et al. 2024).

### 5.5. Nanomicelles carrying chemokines

Polymeric micelles provide a compact and modular nanoplatform for chemokine-axis-guided delivery because amphiphilic polymers or peptide amphiphiles can self-assemble into nanoscale structures with a hydrophobic core and hydrophilic corona, while their surfaces can be functionalized with targeting ligands (Ghezzi et al. 2021). A 23-residue CCL2-derived CCR2-binding peptide was used as a surface-displayed targeting module and combined with the pro-apoptotic KLAKLAK peptide to create a therapeutic peptide micelle (Trac et al. 2021). This design allowed for multivalent presentation of the CCL2-derived CCR2-targeting sequence on the micellar surface, which can enhance receptor binding. Instead of encasing full-length CCL2 as a soluble chemokine cargo, this formulation created a therapeutic peptide micelle by combining the pro-apoptotic KLAKLAK peptide with a 23-residue CCL2-derived CCR2-binding peptide as a surface-displayed targeting module. In comparison to free peptide ligands, this design allows for the multivalent presentation of the CCL2-derived CCR2-targeting sequence on the micellar surface, which can improve receptor binding, shield peptide ligands from serum peptidases, and increase structural stability (Trac et al. 2021).

### 5.6. Microneedle patch carrying chemokines

Compared to traditional injection-based delivery, microneedle patches offer a unique platform for chemokine-associated distribution because they can physically cross tissue barriers, establish local drug or cell depots, and lower systemic exposure (Fan et al. 2026). Zhou et al. created a grooved microneedle patch that addresses the immunosuppressive and physical barriers of solid tumors by combining chemokine-guided Treg-cell diversion with local T-cell delivery (Zhou et al. 2024). Sulfo-SMCC-mediated coupling was used to conjugate CCL22 after the PDA-modified microneedles were treated with Traut's reagent to introduce thiol groups for chemokine immobilization. This surface-anchored CCL22 was able to bind CCR4 on Treg cells while maintaining its bioactivity. Instead of destroying CCR4⁺ Treg cells in situ, this approach employs the microneedle surface as a detachable chemokine-presenting niche that directs them away from the tumor. This is different from soluble chemokine injection. The CCL22-modified grooved microneedle patch considerably inhibited melanoma development, improved tumor death, promoted CD8⁺ T-cell infiltration, decreased FoxP3⁺ Treg-cell accumulation, and extended survival in animal models when paired with TCR-T or CAR-T cells (Zhou et al. 2024).

## 6. Conclusions and perspectives

Chemokines are a fundamental molecular guidance mechanism that shapes the spatial organization and functional state of the TME. Instead of only increasing immune-cell infiltration, chemokine networks regulate the migration of immune cells, their activation or inhibition, and their interactions with tumor, stromal, endothelial, and myeloid compartments. In this review, we find that chemokines can lure anticancer effector cells into tumor cores, attract immunosuppressive populations such as Tregs and TAMs, keep T cells at stromal boundaries, enable immune-cell egress through lymphatic channels, or generate settings that are favorable for metastasis. Therefore, rather than focusing on the simplistic goal of ‘more immune cells in tumors,’ future chemokine-based therapies should aim to replicate the spatial immunological roadmap of tumors.

Instead of being seen as separate therapeutic weapons, chemokines and nanodelivery approaches should be seen as complementary elements. By sheltering chemokines from degradation, extending their local retention, and permitting prolonged or controlled release, nanocarriers can make up for the pharmacological limits of chemokines and aid in the establishment of stable chemokine gradients within tumors. In the TME, these gradients play a critical role in guiding immune-cell recruitment, localization, and functional organization. On the other hand, chemokine receptors on extracellular vesicles, biomimetic nanoparticles, or customized cell membrane-coated carriers can provide nanomedicines with the potential to navigate biologically. These receptor-bearing nanocarriers can increase drug delivery accuracy and tumor homing by detecting and reacting to tumor-associated chemokine gradients. Therefore, while nanocarriers function as stabilizing, localizing, and programmable delivery platforms, chemokines can function as both immunoregulatory cargos and navigational signals. For the development of next-generation chemokine-based nanomedicines for cancer immunotherapy and spatial immune remodeling, this reciprocal interaction provides a crucial framework.

Beyond the direct delivery of chemokine cargos, recent advances in programmable nanomedicine have highlighted the potential of tumor-responsive nanoplatforms to integrate gene editing with immune modulation. For instance, a hierarchical nanoCRISPR system targeting PD-L1 and PCSK9 enhances MHC-I-mediated antigen presentation while concurrently attenuating PD-1/PD-L1-mediated immunosuppression (Li et al. 2025). Similarly, an HO-1-targeting NanoCRISPR scaffold increased tumor sensitivity to reactive oxygen species and, when combined with PD-L1 blockade, induced durable antitumor immunity and immunological memory (Wang et al. 2025). Collectively, these findings suggest that future chemokine-guided nanoplatforms could incorporate gene-editing and immune-resistance-reversal modules to enable more comprehensive remodeling of the tumor immune microenvironment.

Despite these advances, several barriers must be overcome before chemokine-integrated nanomedicines can achieve successful clinical translation. Biosafety remains a major concern, as immunogenicity, unintended biodistribution, degradation products, and dysregulated chemokine release may lead to off-target effects, necessitating systematic evaluation of pharmacokinetics, long-term clearance, and repeated-dose toxicity (Joyce et al. 2024). Reproducibility and large-scale manufacturing are also critical, particularly for multicomponent and biomimetic systems, where batch-to-batch variations in physicochemical properties, surface functionality, cargo loading, and release kinetics may compromise product consistency (Ioannidis et al. 2018). Moreover, inter- and intratumoral heterogeneity can substantially influence nanoparticle accumulation, penetration, retention, and therapeutic response (Mitchell et al. 2021; Bravo et al. 2026). Clinically relevant patient-derived models and biomarker-guided stratification may therefore help optimize formulations for different tumor contexts. Beyond delivering plasmids that regulate chemokine expression, emerging nanotechnologies can directly manipulate chemokine receptor signaling (Shen et al. 2018; Mendez-Gomez et al. 2024; Cheng et al. 2026). For example, CXCR4-targeted nanothreads can promote receptor clustering and remodel CXCR4-mediated metastatic signaling (Zhou et al. 2024). Thus, future chemokine-based nanomedicines should integrate standardized and scalable manufacturing, rigorous biosafety assessment, tumor-specific formulation screening, controlled chemokine release, and receptor-guided navigation or remodeling to improve their clinical robustness and precision.

## Data Availability

Data sharing is not applicable to this article as no new data were created or analyzed in this study.
